# Treatment of iron deficiency in patients scheduled for pancreatic surgery: implications for daily prehabilitation practice in pancreatic surgery

**DOI:** 10.1186/s13741-023-00323-1

**Published:** 2023-07-11

**Authors:** Allard G. Wijma, Michele F. Eisenga, Maarten W. Nijkamp, Frederik J. H. Hoogwater, Joost M. Klaase

**Affiliations:** 1 Department of Surgery, Division of Hepato-Pancreato-Biliary Surgery and Liver Transplantation, University Medical Center Groningen, PO Box 30.001, 9700 RB Groningen, The Netherlands; 2 Department of Internal Medicine, Division of Nephrology, University Medical Center Groningen, PO Box 30.001, 9700 RB Groningen, the Netherlands

**Keywords:** Iron deficiency, Anemia, Pancreatic surgery, Prehabilitation, Preoperative care pathway, Preoperative risk stratification, Patient blood management

## Abstract

**Background:**

Preoperative anemia is a frequent complication in pancreatic surgical patients, and it adversely affects morbidity, mortality, and postoperative red blood cell (RBC) transfusion rates. Iron deficiency (ID) is often the underlying cause of anemia and constitutes a modifiable risk factor.

**Methods:**

Single-center, longitudinal prospective cohort study conducted between May 2019 and August 2022 at the University Medical Center Groningen in the Netherlands. Patients scheduled for pancreatic surgery were referred to the outpatient prehabilitation clinic for preoperative optimization of patient-related risk factors. Patients were screened for anemia (< 12.0 g/dL in women and < 13.0 g/dL in men) and ID (either absolute [ferritin < 30 µg/L] or functional [ferritin ≥ 30 µg/L + transferrin saturation < 20% + C-reactive protein > 5 mg/L]). Intravenous iron supplementation (IVIS) (1,000 mg ferric carboxymaltose) was administered to patients with ID at the discretion of the consulting internist. Pre- and postoperative hemoglobin (Hb) levels were assessed, and perioperative outcomes were compared between patients receiving IVIS (IVIS-group) or standard care (SC-group).

**Results:**

From 164 screened patients, preoperative anemia was observed in 55 (33.5%) patients, and in 23 (41.8%) of these patients, ID was the underlying cause. In 21 patients, ID was present without concomitant anemia. Preoperative IVIS was administered to 25 patients, out of 44 patients with ID. Initial differences in mean Hb levels (g/dL) between the IVIS-group and SC-group at the outpatient clinic and one day prior to surgery (10.8 versus 13.2, *p* < 0.001, and 11.8 versus 13.4, *p* < 0.001, respectively) did not exist at discharge (10.6 versus 11.1, *p* = 0.13). Preoperative IVIS led to a significant increase in mean Hb levels (from 10.8 to 11.8, *p* = 0.03). Fewer SSI were observed in the IVIS-group (4% versus 25.9% in the SC-group, *p* = 0.02), which remained significant in multivariable regression analysis (OR 7.01 (1.68 – 49.75), *p* = 0.02).

**Conclusion:**

ID is prevalent in patients scheduled for pancreatic surgery and is amendable to preoperative correction. Preoperative IVIS increased Hb levels effectively and reduced postoperative SSI. Screening and correction of ID is an important element of preoperative care and should be a standard item in daily prehabilitation practice.

**Supplementary Information:**

The online version contains supplementary material available at 10.1186/s13741-023-00323-1.

## Background

Iron deficiency (ID) is the most common cause of anemia in surgical cancer patients and amendable to preoperative correction (Miles and Richards 2022; Fischer et al. 2019). ID can either present in the form of absolute ID (i.e., a true absence of stored iron) or functional ID (i.e., a pathophysiological condition characterized by inflammation withholding adequate iron stores from the plasma) (Pasricha et al. 2021). Both conditions ultimately result in anemia, and treatment should consist of timely iron supplementation (Miles and Richards 2022). Intravenous iron supplementation (IVIS) is preferred over oral iron supplementation due to fewer gastrointestinal adverse events and better biochemical availability in systemic inflammation conditions, which is often present in oncologic patients (Fischer et al. 2019). Furthermore, unlike oral iron supplementation, IVIS is administered in a single dose, thereby indirectly optimizing patients’ therapy compliance. It is worth noting that ID can present with anemia (IDA) or without concomitant anemia. In both cases, iron supplementation is indicated, since anemia is the end phase of depleted iron stores. Multiple studies have established positive effects of preoperative iron supplementation in surgical patients, with increased hemoglobin (Hb) levels resulting in fewer postoperative RBC transfusions and lower morbidity rates (Triphaus et al. 2021; Froessler et al. 2018; Quinn et al. 2017; Wilson et al. 2018). However, data on preoperative correction of ID with IVIS in patients undergoing pancreatic surgery are lacking. As a result, controversy exists regarding the benefits of preoperative screening for ID and IVIS in case of ID in pancreatic surgery. 

It is pivotal to note that preoperative anemia constitutes a serious and significant modifiable risk factor in surgical cancer patients, with incidence estimates ranging from 25 to 40% (Fowler et al. 2015; Lin 2019). The pathogenesis of anemia in patients with cancer is multifactorial and different mechanisms can coexist. As alluded to above, ID is the most common cause of anemia in cancer patients. Absolute ID can ensue, among others, due to malabsorption and gastrointestinal bleeding, whereas functional ID can typically be present due to the systemic inflammation present in cancer patients. Malabsorption can also lead to other anemia-contributing nutritional deficiencies. The latter might also be caused by exocrine pancreas insufficiency especially in pancreatic cancer. Finally, malnutrition and metastatic infiltration of bone marrow often contribute to anemia in cancer patients (Abiri and Vafa 2020; Busti et al. 2018 Vujasinovic et al. 2017). Preoperative anemia, even to a mild degree, is associated with a significant increase in postoperative morbidity and mortality (Musallam et al. 2011; Beattie et al. 2009; Oehme et al. 2021). To illustrate, in a cohort study investigating the consequences of preoperative anemia in patients undergoing major noncardiac surgery, compared with patients without anemia, patients with anemia were found to have higher rates of almost all specific morbidities (e.g., cardiac, respiratory, wound events, sepsis) (Musallam et al. 2011). Moreover, when left untreated, preoperative anemia is associated with an increased requirement for postoperative red blood cell (RBC) transfusions (Fowler et al. 2015; Beattie et al. 2009; Luo et al. 2020; Pecorelli et al. 2022). In turn, postoperative RBC transfusions have been suggested to adversely affect cancer treatment outcomes, with reduced disease-free and overall survival (Wu et al. 2018; Acheson et al. 2012; Schiergens et al. 2015). Additionally, RBC transfusions are expensive and contribute substantially to hospital costs (Shander et al. 2010). It is, therefore, a misconception to consider preoperative anemia a relatively harmless condition for which RBC transfusion is an efficient treatment when symptomatic.

To substantiate the routine use of IVIS in pancreatic surgical patients, we investigated its effect on increasing perioperative Hb levels and improving postoperative outcomes in patients with ID and IDA.

## Methods

### Study design and setting

This single-center, longitudinal prospective cohort study was conducted between May 2019 and August 2022 at the University Medical Center Groningen in the Netherlands and is part of the Frail-study (Wijk et al. 2021). In the Frail-study patients are screened and assessed for modifiable patient related risk-factors. In the current study, the effect of preoperative IVIS was investigated by comparing patients receiving IVIS (IVIS-group) to patients receiving standard care (SC-group) prior to pancreatic resection. All included patients completed the informed consent process, which was approved by the Institutional Review Board of the University Medical Center Groningen (Netherlands research registration number 201800293). This study was performed in accordance with the ethical standards set by the Declaration of Helsinki.

### Patient inclusion

All consecutive patients scheduled for elective pancreatic resection at the University Medical Center Groningen aged 18 years or older and who visited our prehabilitation outpatient clinic preoperatively were included in this study. Patients with missing preoperative laboratory results or who underwent palliative surgery instead of a surgical resection (e.g., due to metastasis or irresectable disease) were excluded from the final analysis.

### Perioperative care

As part of the Frail-study, all patients scheduled to undergo hepatopancreatobiliary surgery are referred to the prehabilitation outpatient clinic to screen for patient related modifiable risk-factors. The prehabilitation outpatient clinic visit is integrated in the preoperative work-up of patients. The method of screening and assessment at the prehabilitation outpatient clinic has been published previously (Wijk et al. 2021). In summary, physical fitness of patients is evaluated, and in case of low physical fitness, patients are advised to participate in an exercise program to improve their physical fitness prior to surgery. Furthermore, a specialist dietician screens patients for malnutrition and provides them with dietary advice and/or nutritional supplements and pancreatic enzyme replacement therapy (PERT). Moreover, screening focuses on hyperglycemia, (causes of) anemia, frailty, substance abuse (e.g., smoking, alcohol consumption), and mental resilience. Based on screening results, interventions are employed as appropriate. Perioperative care in our hospital is in accordance with the latest Enhanced Recovery after Surgery protocol for pancreatic surgery (Melloul et al. 2020). To prevent surgical site infections (SSI), cutaneous chlorhexidine gluconate disinfectant is used intraoperatively, and preoperative intravenous antibiotic prophylaxis (Cefazoline) is administered to all patients. Additionally, in patients with a biliary stent Fluconazole is administered preoperatively.

### Assessment of iron deficiency (anemia)

To assess whether patients suffered from ID or IDA, Hb, ferritin, iron, transferrin, transferrin saturation (TSAT), and C-reactive protein (CRP) were standard items in the preoperative laboratory screening. The Hb level was measured using a Sysmex XN10 analyzer (Sysmex Corp., Kobe, Japan). Furthermore, serum iron was measured using a colorimetric assay, ferritin was measured using immunoassay, and transferrin was measured using an immunoturbidimetric assay (Roche Diagnostics, Mannheim, Germany). TSAT was calculated as 100 × serum iron (μmol/L) / (25.2 × transferrin (g/L)). Anemia was defined as an Hb level of < 12.0 g/dL in women and < 13.0 g/dL in men (WHO 2011). ID was defined as either absolute (ferritin < 30 µg/L) or functional (ferritin ≥ 30 µg/L + TSAT < 20% + CRP ≥ 5.0 mg/L). Because ferritin is a well-known acute-phase reactant, a higher cutoff value for functional ID was chosen compared to absolute ID (Kernan and Carcillo 2017). Moreover, since controversy exists regarding the upper limit of ferritin (ranging up to 800 µg/L) in association with TSAT < 20% at which iron should be prescribed in functional ID, we chose to refer patients for IVIS to the internal medicine department when matching any of these criteria (Busti et al. 2018). Ultimately, the decision to refer a patient for IVIS was at the discretion of the consulting surgeon and final approval for IVIS was given by the consulting internist. All patients in the IVIS-group received a single dose of 1,000-mg intravenous ferric carboxymaltose preoperatively to elevate serum iron and Hb levels (Munting and Klein 2019). In case of anemia in the absence of ID, alternative causes of anemia were investigated (e.g., folate or vitamin B-12 deficiency) and treated appropriately.

### Data collection and study endpoints

Patient characteristics, laboratory results, time between IVIS and surgery, and clinical outcome data were collected from patients’ electronic medical records. The primary endpoint of this study was the change in Hb levels over time. For this, blood was collected from patients during the first prehabilitation outpatient clinic visit (T0), at admission (T1), and at discharge (T2). Iron parameters (i.e., ferritin, iron, transferrin, and TSAT) were solely evaluated during the outpatient clinic visit. The secondary endpoints included the need for postoperative RBC transfusion, surgery-specific postoperative complications up to 30 days after surgery, length of hospital stay, and unplanned readmission rate. RBC transfusion was defined as any allogenic RBC transfusion during the postoperative hospital stay. Postoperative pancreatic fistula (POPF), post pancreatectomy hemorrhage (PPH), delayed gastric emptying (DGE), bile leakage (BL), and postoperative chyle leakage (CL) were defined and graded according to ISGPS and ISGLS classifications (Bassi et al. 2017; Koch et al. 2011). SSI were defined as either superficial (cutis and subcutis), deep tissue (fascia and muscle), or organ space (abdominal cavity) infections.

### Statistical analysis

The normality of continuous data was checked using the Shapiro–Wilk test and QQ-plots. Continuous variables are presented as mean with standard deviation (SD) or as median with interquartile range (IQR) based on normality of distribution. Categorical data are presented as numbers and percentages. Differences between groups were calculated using the student’s t-test, Mann–Whitney U test, Chi-squared test, or Fisher exact test, as appropriate. Furthermore, mean differences in specific follow-up points of Hb levels between groups were determined using one-way ANOVA and Tukey’s range test. Multivariable logistic regression analysis with stepwise backward elimination was performed to assess the effect of iron supplementation on the occurrence of SSI. The eligible variables for the adjusted model were selected when the univariable analysis yielded a *p*-value of less than 0.10, or when variables were theoretically considered clinically relevant for the occurrence of SSI. Effect modification by smoking was also tested by including an interaction term (i.e., treatment group x smoking). All models yielded an estimated regression coefficient (β), with a corresponding 95% confidence interval for the hazard ratio and odds ratio with 95% confidence interval. The R software package, version 4.2.2. (R Foundation for Statistical Computing, Vienna, Austria) was used for statistical analysis, using the ggpubr, tidyverse, and ggplot2 packages. In all analyses, a *p*-value < 0.05 was considered statistically significant.

## Results

### Incidence of preoperative ID(A) and IVIS referrals

**Fig. 1:**
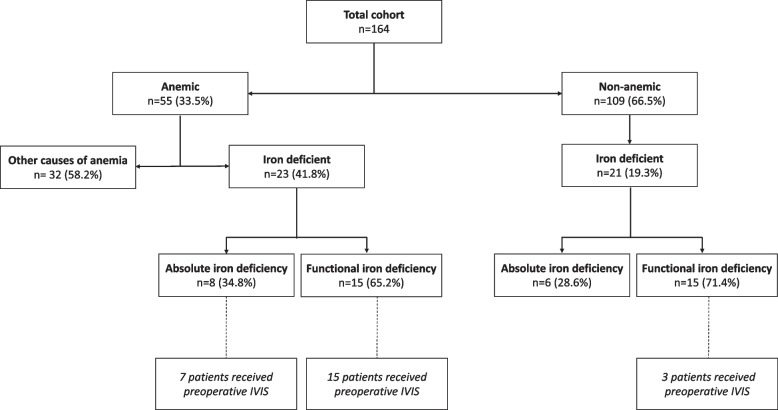
General overview of preoperative anemia and iron deficiency in 164 pancreatic surgical patients. Abbreviations: IVIS = intravenous iron supplementation

A total of 164 patients were analyzed for ID and IDA, of whom 25 were referred for IVIS (Fig. 1). As can been concluded from Fig. 1, not all patients with ID were referred for IVIS. First, one patient with absolute IDA did not receive preoperative IVIS. Although this patient was referred for IVIS, the time between IVIS and surgery was considered too short to achieve a clinically sufficient effect of IVIS. Furthermore, in the nonanemic group, some patients with ID (6 absolute ID and 12 functional ID) did not receive IVIS. This was either for the reason that ID was not noticed in these patients or it was considered not necessary due to adequate Hb levels. The median time between IVIS and surgery was 15 (8–35) days.

### Patient characteristics

The patient characteristics for both study groups are presented in Table 1. Patients in the IVIS-group were slightly older (mean 69.6 years versus 66.1 years in the SC-group, *p* = 0.08), predominantly female (72% versus 55.4% in the SC-group, *p* = 0.12), and had a mean BMI of 27.5 kg/m^2^ (versus 25.9 kg/m^2^ in the SC-group, *p* = 0.15). The percentage of patients with an ASA classification 3 was higher in the IVIS-group (52% versus 30.2% in the SC-group, *p* = 0.03). In contrast, the incidence of tobacco abuse was higher in the SC-group (30.2% versus 4% in the IVIS-group, *p* < 0.01). Other relevant patient characteristics were well balanced between groups.

**Table 1:** Characteristics of patients receiving IVIS and standard care

	**IVIS-group** ***n* = 25 (15.2%)**	**SC-group** ***n* = 139 (84.8%)**	***p*-value**
**Mean age: years**	69.6 ± 9.0	66.1 ± 9.2	0.08
**Female gender**	18 (72)	77 (55.4)	0.12
**Mean BMI: kg/m**^**2**^	27.5 ± 5.7	25.9 ± 4.8	0.15
**Charlson comorbidity index ≥ 4**	10 (40)	60 (43.2)	0.77
**ASA classification ≥3**	13 (52)	42 (30.2)	**0.03**
**Medical history**			
Diabetes Mellitus	3 (12)	32 (23)	0.22
Hypertension	13 (52)	62 (44.6)	0.49
Heart disease	3 (12)	14 (10.1)	0.77
Respiratory disease	1 (4)	17 (12.2)	0.31
**Substance abuse**			
Tobacco	1 (4)	42 (30.2)	**0.005**
Alcohol	11 (44)	68 (48.9)	0.65
**Neoadjuvant treatment**	4 (16)	14 (10.1)	0.48
**Preoperative biliary decompression**	9 (36)	60 (43.2)	0.50

Data are presented as mean ± standard deviation, median (IQR), or number (%)

*Abbreviations: IVIS* Intravenous iron supplementation, *SC* Standard care, *BMI* Body mass index, *ASA* American Society of Anesthesiologists’ score

### Laboratory results

**Fig. 2:**
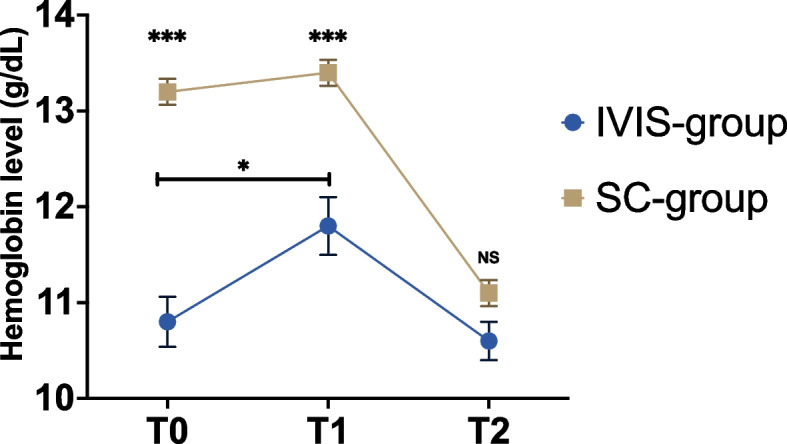
Course of Hb levels (g/dL) over the perioperative period in the IVIS- and SC-group. Abbreviations: IVIS = intravenous iron supplementation; SC = standard care; T0 = outpatient clinic; T1 = admission; T2 = discharge. *p < 0.05; ***p < 0.001; NS = not significant

As expected, preoperative iron parameters were significantly lower in the IVIS-group, with a ferritin level of 88 μg/L versus 216 μg/L (*p* < 0.001) and TSAT 12.9% versus 25.9% (*p* < 0.001) in the IVIS- and SC-group, respectively (Table 2). Consequently, a significantly lower preoperative mean Hb level was observed in the IVIS-group (10.8 g/dL versus 13.2 g/dL in the SC-group, *p* < 0.001). This difference in mean Hb level remained statistically significant at admission (11.8 g/dL versus 13.4 g/dL in the IVIS-group and SC-group, respectively, *p* < 0.001) yet no longer existed at discharge (10.6 g/dL versus 11.1 g/dL in the IVIS-group and SC-group, respectively, *p* = 0.13) (Table 2). In Fig. 2, between group differences in serum Hb levels over time are displayed, demonstrating a significant increase in mean Hb level as a result of IVIS in the IVIS-group between outpatient clinic and admission (10.8 g/dL and 11.8 g/dL, respectively, *p* = 0.03). It is worth noting that in both groups, a significant decrease in mean Hb level occurred between admission and discharge (from 11.8 g/dL to 10.6 g/dL in the IVIS-group, *p* = 0.01, and 13.4 g/dL to 11.1 g/dL in the SC-group, *p* < 0.001).

**Table 2:** Detailed overview of anemia and iron parameters in patients receiving IVIS and standard care

	**IVIS-group** ***n* = 25 (15.2%)**	**SC-group** ***n* = 139 (84.8%)**	***p*-value**
**Outpatient clinic laboratory results**			
Hemoglobin: g/dL	10.8 ± 1.3	13.2 ± 1.6	** < 0.001**
Ferritin: μg/L	88 (29–190)	216 (104–448)	** < 0.001**
TSAT: %	12.9 ± 4.9	25.9 ± 12.8	** < 0.001**
**Admission laboratory results**			
Hemoglobin: g/dL	11.8 ± 1.5	13.4 ± 1.6	** < 0.001**
**Discharge laboratory results**			
Hemoglobin: g/dL	10.6 ± 1	11.1 ± 1.6	0.13

Data are presented as mean ± standard deviation or as median (IQR)

*Abbreviations: IVIS* Intravenous iron supplementation, *SC* Standard care, *TSAT* Transferrin saturation

### Surgical details and postoperative outcomes

The types of pancreatic resections and rate of complementary (vascular) resection was similar between groups (Table 3). Albeit not statistically significant, the median intraoperative blood loss was higher in the IVIS-group (500 mL versus 400 mL in the SC-group, *p* = 0.07). Also, the rate of RBC transfusions administered in the IVIS-group was slightly higher (36% versus 20.9% in the SC-group, *p* = 0.10). The median length of hospital stay was 12 days versus 11 days in the IVIS-group and SC-group, respectively, *p* = 0.23. The rate of surgery-specific complications and cardiopulmonary complications did not differ between groups. In the SC-group, significantly more SSI (31 superficial and 5 deep tissue SSI versus 1 superficial SSI in the IVIS-group) were observed (25.9% versus 4% in the IVIS-group, *p* = 0.02). This effect remained significant when tested (*p*-value not significant) for the higher tobacco abuse incidence in the SC-group in a multivariable logistic regression analysis (Table 4). Effect modification by smoking was not observed in the multivariable logistic regression model.

**Table 3:** Surgical details and postoperative outcomes

	**IVIS-group** ***n* = 25 (15.2%)**	**SC-group** ***n* = 139 (84.8%)**	***p*-value**
**Surgical procedure**			0.79
Pancreatoduodenectomy	20 (80)	107 (77)	
Distal pancreatectomy	3 (12)	23 (16.5)	
Other	2 (8)	9 (6.5)	
**Complementary resection**	5 (20)	27 (19.4)	0.95
**Vascular resection**			
Arterial	0	6 (4.3)	0.59
Venous	4 (16)	28 (20.1)	0.79
**Intraoperative blood loss: ml**	500 (400–800)	400 (250–737.5)	0.07
**Length of hospital stay: days**	12 (9–20)	11 (8–17)	0.23
**Surgery specific complications**			
POPF ≥ grade B	4 (16)	27 (19.4)	0.79
DGE ≥grade B	6 (24)	30 (21.6)	0.79
BL ≥ grade B	1 (4)	3 (2.2)	0.49
PPH ≥ grade B	2 (8)	9 (6.5)	0.68
CL ≥ grade B	4 (16)	12 (8.6)	0.27
**SSI**	1 (4)	36 (25.9)	**0.02**
**RBC transfusion**	9 (36)	29 (20.9)	0.10
**In-hospital mortality**	1 (4)	3 (2.2)	0.49
**Unplanned readmission < 30 days**	4 (16)	18 (12.9)	0.75

Data are presented as median (IQR) or number (%)

*Abbreviations: IVIS* Intravenous iron supplementation, *SC* Standard care, *POPF* Postoperative pancreatic fistula, *DGE* Delayed gastric emptying, *BL* Bile leakage, *PPH* Post-pancreatectomy hemorrhage, *CL* Chyle leakage, *SSI* Surgical site infections, *RBC* Red blood cell, *ICU* Intensive care unit

**Table 4:** Results of the multivariable logistic regression analysis

Outcome	Predictor	β (95% CI)	Odds ratio (95% CI)	*p*-value
SSI^a^	Standard care	1.95 (0.52 – 3.91)	7.01 (1.68 – 49.75)	**0.02**
	Age	-0.06 (-0.10 – -0.01)	0.95 (0.90 – 0.99)	**0.01**
	ASA classification ≥ 3	1.04 (0.19 – 1.91)	2.83 (1.21 – 6.78)	**0.02**
	RBC transfusion	1.42 (0.54 – 2.32)	4.15 (1.73 – 10.17)	**0.002**

Tested: age, gender, BMI, ASA classification ≥ 3, tobacco use, Intraoperative blood loss, RBC transfusion

*Abbreviations: β *Beta, *CI* Confidence interval, *SSI* Surgical site infections

^a^adjusted for age, ASA classification ≥ 3, RBC transfusion

### Subgroup analysis

Results of a subgroup analysis comparing the IVIS-group to the nontreated ID-group is available in the supplementals. The mean age in this subgroup analysis was higher in the IVIS-group (69.6 years versus 62.4 years in the nontreated ID-group, *p* = 0.04) (Table S1). Moreover, the incidence of tobacco abuse was again lower in the IVIS-group (4% versus 42.1% in the nontreated ID-group, *p* = 0.003). When comparing the laboratory results (Table S2), it becomes clear that, despite similar iron parameters between groups, in the nontreated ID-group, fewer patients had preoperative anemia, with a mean Hb level at the outpatient clinic of 13.5 g/dL (versus 10.8 g/dL in the IVIS-group, *p* < 0.001) and 13.0 g/dL at admission (versus 11.8 g/dL in the IVIS-group, *p* = 0.002). However, while the preoperative Hb level significantly increased after IVIS in the IVIS-group, in the nontreated ID-group, a decline in Hb level was observed (from 13.5 g/dL to 13.0 g/dL, *p* = 0.55). At discharge, Hb levels between groups were comparable (10.6 g/dL and 10.9 g/dL in the IVIS- and non-treated ID-group, respectively, *p* = 0.36). In the nontreated ID-group, the incidence of POPF (47.4% versus 16% in the IVIS-group, *p* = 0.04) and SSI (36.8% versus 4% in the IVIS-group, *p* = 0.01) was significantly higher.

## Discussion

In this study, we demonstrated an important role of preoperative IVIS in pancreatic surgery patients with IDA and ID. Preoperative anemia was present in more than 30% of the patients, and in more than 40% of these patients, ID was the underlying cause of anemia. In around 20% of the nonanemic patients, ID was present, making these patients also susceptible for anemia. In 25 patients, preoperative IVIS led to a significant increase in mean preoperative Hb level (from 10.8 g/dL to 11.8 g/dL), which resulted in an almost equal mean Hb level at discharge compared to patients who did not receive preoperative IVIS (10.6 g/dL versus 11.1 g/dL, respectively), suggesting high effectiveness of preoperative IVIS. This preoperative lift in mean Hb levels might have enhanced postoperative outcomes in these patients, resulting in similar surgery-specific complication rates compared to patients without IDA. Moreover, a clinically relevant reduction in SSI was observed in the IVIS-group. No differences in postoperative RBC transfusions were observed. However, postoperative RBC transfusion demand is greatly influenced by the amount of intraoperative blood loss, and the higher median intraoperative blood loss in the IVIS-group will have had a clinically relevant impact on transfusion demands.

Our results are supported by previous studies demonstrating the beneficial effect of preoperative IVIS on increasing preoperative Hb levels in various surgical populations (Triphaus et al. 2021; Froessler et al. 2018; Quinn et al. 2017; Wilson et al. 2018; Janssen et al. 2021). As illustrated in the study of Triphaus et al., the extent to which the Hb level increases preoperatively depends mainly on the timing of the preoperative IVIS (Triphaus et al. 2021). The maturation from erythroblast to proliferated RBC is an iron-dependent process and takes up to 4–6 days (Triphaus et al. 2021; Besarab et al. 2009) Consequently, a therapeutic effect of IVIS can be expected after 5–7 days and maximal increase of Hb levels after 4–6 weeks. (Triphaus et al. 2021; Besarab et al. 2009) Considering the generally narrow time span before cancer surgery, it will not always be feasible to reach maximal increase of Hb levels. Nevertheless, previous results indicate a clinically relevant effect can be expected when IVIS is given at least seven days before surgery (Triphaus et al. 2021). In our cohort, the median time between IVIS and surgery was 15 (8–35) days, resulting in a 1.0 g/dL increase in mean preoperative Hb level. However, we did not observe a difference in postoperative RBC transfusion rates between groups. Taking into account that, in this study, predominantly anemic patients were compared to nonanemic patients, a reduction in postoperative RBC transfusion rate might be expected when analyzing solely anemic patients with ID. To underpin this theory, multiple studies have successfully demonstrated a reduction in postoperative RBC transfusion demand after preoperative IVIS (Triphaus et al. 2021; Froessler et al. 2018; Quinn et al. 2017). RBC transfusions in oncologic patients are also associated with adverse treatment outcomes and should therefore be avoided when possible (Wu et al. 2018; Acheson et al. 2012; Schiergens et al. 2015). When dealing with anemia in patients, one should restore or maintain adequate iron storages, thereby enabling the physiological ability to restore normal Hb levels, and administer RBC transfusions with restraint.

### Iron and prehabilitation

Iron plays an essential role in oxidative energy production, and it is most known for its function in the erythropoiesis and formation of Hb, thereby facilitating oxygen transport (Besarab et al. 2009; Yiannikourides and Latunde-Dada 2019; Haas and Brownlie 2001). ID will impair these processes, eventually resulting in anemia (Haas and Brownlie 2001). However, it is a misconception to solely focus on anemia as a consequence of ID, since iron has multiple important roles in maintaining physiological homeostasis. To demonstrate, iron also plays a crucial role in the synthesis of myoglobin, an oxygen storage protein in muscle tissue capable of releasing oxygen during hypoxia (Yiannikourides and Latunde-Dada 2019; Ordway and Garry 2004). Importantly, ID is accompanied by reduced availability of myoglobin, resulting in tissue remodeling and impaired organ efficacy (e.g., the heart muscle) (Stugiewicz et al. 2016; Jankowska et al. 2013). Furthermore, iron facilitates energy metabolism at the cellular level in mitochondria, and ID will result in mitochondrial dysfunction, for which cells with a high energy demand (e.g., skeletal and cardiac myocytes) are particularly sensitive. (Yiannikourides and Latunde-Dada 2019; Haas and Brownlie 2001; Jankowska et al. 2013). As a consequence of the aforementioned iron-related mechanisms, ID, with or without concomitant anemia, has been found to be associated with impaired exercise tolerance (Anker et al. 2009a; Jankowska et al. 2011; Elezaby et al. 2021; Martens et al. 2021). To illustrate, in the study of Jankowska et al., patients with chronic heart failure (CHF), both with and without ID, were subjected to cardiopulmonary exercise testing (CPET) (Jankowska et al. 2011). They found that ID was independently associated with a significant reduction in peak oxygen consumption (VO_2_peak) (Jankowska et al. 2011). Moreover, in the study of Martens et al. subjecting patients with unexplained dyspnea to a CPET-echo, it was found that ID was independently associated with a lower VO_2_peak and maximal workload (WR_peak_) and reduced cardiac output reserve during exercise, resulting in diminished exercise capacity (Martens et al. 2021). In patients with CHF and ID, IVIS (ferric carboxymaltose) was found to be beneficial for both patients with and without anemia and led to significant improvements in Hb levels, the distance on the 6-min walk test, and quality-of-life assessments (Anker et al. 2009b). The effect of ID on exercise tolerance is also highly relevant in surgical patients, since impaired exercise tolerance is associated with adverse postoperative outcomes (Junejo et al. 2014; Wilson et al. 2010). ID should perhaps be seen as a separate condition from anemia, independently adversely affecting energy metabolism resulting in diminished exercise capacity, and should therefore be treated accordingly, regardless of the presence of concomitant anemia.

In our cohort, we observed significantly fewer SSI in the IVIS-group. When correcting for confounders (e.g., tobacco abuse), this effect remained significant. Moreover, iron supplementation might have had a beneficial effect. Wound tissue macrophages play an essential role in effective wound healing by orchestrating tissue repair (Wilkinson et al. 2019a). At the site of injury, macrophages undergo marked morphologic and behavioral changes, and it is hypothesized that iron plays an important role in modulating macrophage behavior to promote healing (Wilkinson et al. 2019a; Wilkinson et al. 2019b). Experimental studies revealed an accumulation of iron in later stages of wound healing, and this was linked to increased macrophage differentiation (Wilkinson et al. 2019a; Wilkinson et al. 2019b). Therefore, increased availability of serum iron might promote wound healing.

The results from our study point out the potential of iron supplementation in pancreatic surgical patients with preoperative ID. However, some patients with ID without concomitant anemia did not receive preoperative IVIS in our cohort. Especially in patients with functional ID, there is a certain reluctance to prescribe IVIS due to the fact that systemic inflammation is a frequent finding in cancer patients, which might perturb iron measurements (McSorley et al. 2016). However, functional ID is the predominant mechanism in cancer patients and results in reduced iron availability (Miles and Richards 2022; Naoum 2016). IVIS has been found to overcome the absorptive inflammatory blockade of iron, effectively increasing iron stores (Naoum 2016). Previously, concerns regarding the safety of iron supplementation in cancer patients existed, since iron serves as an important growth factor in rapidly differentiating cells, including tumor cells. Nevertheless, studies suggest IVIS is safe in cancer patients and is not associated with adverse events (Lebrun et al. 2017; Gilreath et al. 2012).

The results of this study must be interpreted in light of some limitations. Its first limitation lies in the fact that this was a single-center study with an observational design, impeding the generalizability of these results. Due to its observational design with a pragmatic approach, treating physicians’ therapy adherence was not carefully monitored, and subsequently, not all patients with IDA or ID received preoperative IVIS. This unintentional selection bias influenced the results of the SC-group, skewing the data due to patients with ID and IDA in this group. Based on the results of this study, no decisive conclusions on the effect of IVIS can be drawn. However, the marked increase in mean Hb level in the IVIS-group, the primary outcome of this study, is indisputably an effect of preoperative IVIS. Furthermore, we performed a subgroup analysis, comparing the IVIS-group to the nontreated ID-group, which yielded similar results. Since only 30-day follow-up data was available for analysis, the long-term effects of IVIS on patient outcomes (e.g., on mortality rates) could not be investigated. Finally, the effect of IVIS on restoring iron deposits was not evaluated, as iron parameters were only assessed at the outpatient clinic. Moreover, this study lacked other relevant iron parameters (e.g., hepcidin, reticulocyte hemoglobin equivalent) which can adequately assess ID and restoration of iron deposits in red blood cells in inflammatory conditions. Nevertheless, a clinically adequate increase in mean Hb levels was observed in this study. For future studies, ideally a randomized controlled trial with an adequate sample size comparing the effect of IVIS versus standard care in two ID patient cohorts would be the preferred study design.

In conclusion, the results of this study demonstrate a high incidence of ID in pancreatic surgical patients and the potential of preoperative IVIS to increase mean Hb level. ID constitutes a significant risk factor in patients, which is amendable to preoperative correction. In addition to increasing mean Hb levels, IVIS led to a reduction in postoperative SSI. However, as we observed in this study, protocol adherence to refer patients for preoperative IVIS is suboptimal. We have established that ID is a serious issue in pancreatic surgical patients, and therefore we conclude that preoperative screening and correction of ID are important elements of preoperative care and should be standard items in daily prehabilitation practice.

## Supplementary Information

**Additional file 1: Table S1. **Characteristics of patients receiving IVIS and patients with nontreated ID. **Table S2. **Detailed overview of anemia and iron parameters in patients receiving IVIS and patients with nontreated ID. **Table S3. **Surgical details and postoperative outcomes.

## Data Availability

The datasets generated or analyzed in the present study are not publicly available because the data are linked to a vulnerable patient population. However, these data are available from the corresponding author (a.g.wijma@umcg.nl) upon reasonable request.

## Abbreviations

RBC: Red blood cell
ID: Iron deficiency
IVIS: Intravenous iron supplementation
IDA: Iron deficiency anemia
Hb: Hemoglobin
SC: Standard care
PERT: Pancreatic enzyme replacement therapy
TSAT: Transferrin saturation
CRP: C-reactive protein
POPF: Postoperative pancreatic fistula
PPH: Post pancreatectomy hemorrhage
DGE: Delayed gastric emptying
BL: Bile leakage
CL: Chyle leakage
SSI: Surgical site infections
SD: Standard deviation
IQR: Interquartile range
CHF: Chronic heart failure
CPET: Cardiopulmonary exercise testing
VO_2_peak: Peak oxygen consumption
WR_peak_: Maximal workload
