# Supplementary material for: Treatment of iron deficiency in patients scheduled for pancreatic surgery: implications for daily prehabilitation practice in pancreatic surgery
Source: Perioper Med (Lond). 2023 Jul 11;12:36. doi: 10.1186/s13741-023-00323-1 (PMC10337134; doi:10.1186/s13741-023-00323-1)
Supplement: Supplementary file 1 — Additional file 1: Table S1. Characteristics of patients receiving IVIS and patients with nontreated ID. Table S2. Detailed overview of anemia and iron parameters in patients receiving IVIS and patients with nontreated ID. Table S3. Surgical details and postoperative outcomes. [file 13741_2023_323_MOESM1_ESM.docx]

**Supplementals**

**Table S1.**

**Title:** Characteristics of patients receiving IVIS and patients with nontreated ID.

|  | IVIS-group  n=25 (56.8%) | Non-treated ID-group  n=19 (43.2%) | *p*-value |
| --- | --- | --- | --- |
| Mean age: years | 69.6 ± 9.0 | 62.4 ± 13.0 | **0.04** |
| Female gender | 18 (72) | 13 (68.4) | 0.80 |
| Mean BMI: kg/m^2^ | 27.5 ± 5.7 | 26.6 ± 3.6 | 0.54 |
| Charlson comorbidity index ≥4 | 10 (40) | 7 (36.8) | 0.83 |
| ASA classification ≥3 | 13 (52) | 7 (36.8) | 0.32 |
| Medical history  Diabetes Mellitus  Hypertension  Heart disease  Respiratory disease | 3 (12)  13 (52)  3 (12)  1 (4) | 3 (15.8)  8 (42.1)  3 (12)  1 (5.3) | 1.00  0.52  1.00  1.00 |
| Substance abuse  Tobacco  Alcohol | 1 (4)  11 (44) | 8 (42.1)  9 (47.3) | **0.003**  0.82 |
| Neoadjuvant treatment | 4 (16) | 0 | 0.12 |

**Legend:** Data are presented as mean ± standard deviation, median (IQR), or number (%).

**Abbreviations:** IVIS = intravenous iron supplementation; SC = standard care; BMI = body mass index; ASA = American Society of Anesthesiologists’ score.

**Table S2.**

**Title:** Detailed overview of anemia and iron parameters in patients receiving IVIS and patients with nontreated ID.

|  | IVIS-group  n=25 (56.8%) | Non-treated ID-group  n=19 (43.2%) | *p*-value |
| --- | --- | --- | --- |
|  |  |  |  |
| Outpatient clinic laboratory results  Hemoglobin: g/dL  Ferritin: μg/L  Transferrin saturation: % | 10.8 ± 1.3  88 (29-190)  12.9 ± 4.9 | 13.5 ± 1.1  47 (23.5-207)  15.0 ± 5.3 | **<0.001**  0.82  0.18 |
| Admission laboratory results  Hemoglobin: g/dL | 11.8 ± 1.5 | 13.0 ± 1.1 | **0.002** |
| Discharge laboratory results  Hemoglobin: g/dL | 10.6 ± 1 | 10.9 ± 1.1 | 0.36 |

**Legend:** Data are presented as mean ± standard deviation or as median (IQR).

**Abbreviations:** IVIS = intravenous iron supplementation; SC = standard care; TSAT = transferrin saturation.

**Table S3.**

**Title:** Surgical details and postoperative outcomes.

|  | IVIS-group  n=25 (56.8%) | Non-treated ID-group  n=19 (43.2%) | *p*-value |
| --- | --- | --- | --- |
| Surgical procedure  Pancreatoduodenectomy  Distal pancreatectomy  Other | 20 (80)  3 (12)  2 (8) | 12 (63.2)  5 (26.3)  2 (10.5) | 0.42 |
| Complementary resection | 5 (20) | 4 (21.1) | 1.00 |
| Vascular resection  Arterial  Venous | 0  4 (16) | 1 (5.3)  1 (5.3) | 0.43  0.37 |
| Intraoperative blood loss: ml | 500 (400-800) | 500 (200-675) | 0.26 |
| Length of hospital stay: days | 12 (9-20) | 19 (9.5-29.5) | 0.45 |
| Surgery specific complications  POPF ≥ grade B  DGE ≥ grade B  BL ≥ grade B  PPH ≥ grade B  CL ≥ grade B | 4 (16)  6 (24)  1 (4)  2 (8)  4 (16) | 9 (47.4)  9 (47.4)  1 (5.3)  3 (15.8)  0 | **0.04**  0.11  1.00  0.64  0.12 |
| SSI | 1 (4) | 7 (36.8) | **0.01** |
| RBC transfusions | 9 (36) | 4 (21.1) | 0.34 |
| In-hospital mortality | 1 (4) | 0 | 1.00 |
| Unplanned readmission <30 days | 4 (16) | 5 (26.3) | 0.47 |

**Legend:** Data are presented as median (IQR) or number (%).

**Abbreviations:** IVIS = intravenous iron supplementation; SC = standard care; POPF = postoperative pancreatic fistula; DGE = delayed gastric emptying; BL = bile leakage; PPH = post-pancreatectomy hemorrhage; CL = chyle leakage; RBC = red blood cell; ICU = intensive care unit.
